# Toward more reliable stability measurements in stance: recommendations for number of measurements, foot position and feedback -- a cross-sectional study among servicemen

**DOI:** 10.1186/s40779-019-0212-y

**Published:** 2019-07-12

**Authors:** Saskia Maria Theresia van der Heijden, Maarten Reinders Prins, Peter van der Wurff

**Affiliations:** 1 Physical Therapy Sciences, Program in Clinical Health Sciences, University Medical Center Utrecht, 3584CX Utrecht, The Netherlands; 2 Research and Development, Military Rehabilitation Center Aardenburg, Korte Molenweg 3, 3941PW Doorn, The Netherlands; 3 Department of Human Movement Sciences, Faculty of Behavioural and Movement Sciences, Vrije Universiteit Amsterdam and Amsterdam Movement Sciences, 1081BT Amsterdam, The Netherlands; 4 Institute for Human Movement Studies, HU University of Applied Sciences Utrecht, 3584CS Utrecht, The Netherlands

**Keywords:** Postural stability, Center of Pressure, Servicemen, Intrasession reliability, Concurrent validity

## Abstract

**Background:**

In the military, insufficient postural stability is a risk factor for developing lower extremity injuries. Postural stability training programs are effective in preventing these injuries. However, an objective method for the measurement of postural stability in servicemen is lacking. The primary objective of this study was to assess the influence of the number of repetitions, different foot positions and real-time visual feedback on postural stability, as well as their effects on the intrasession reliability of postural stability measurements in servicemen. The secondary objective was to assess the concurrent validity of the measurements.

**Methods:**

Twenty healthy servicemen between 20 and 50 years of age and in active duty were eligible for this quantitative, cross-sectional study. The measurements took place on a force plate, measuring the mean velocity of the center of pressure. The participants were asked to stand as still as possible in three different foot positions (wide stance, small stance, and on one leg), five times each for 45 s each time, and the measurements were performed with and without real-time visual feedback.

**Results:**

We observed a significant main effect of foot position (*P* < 0.001), but not of visual feedback (*P* = 0.119) or repetition number (*P* = 0.915). Postural stability decreased in the more challenging foot positions.

The ICC estimates varied from 0.809 (one repetition in wide stance) to 0.985 (five repetitions on one leg). The common variance (*R*^2^) between different foot positions without feedback varied between 0.008 (wide stance) and 0.412.

**Conclusions:**

To yield reliable data, wide-stance measurements should be conducted three times, and small-stance measurements and measurements on one leg should be conducted two times.

The scores of a measurement in a particular foot position cannot predict the scores of measurements in other foot positions.

## Background

Sufficient postural stability of the lower extremity (LE) is essential for servicemen to perform operational tasks and ceremonial activities [1–4]. Postural instability increases the risk of LE injuries in the military, as does improper footwear and heavy equipment [5–7].

Among servicemen, injuries of the LE account for approximately 40% of all injuries, leading to high costs, disability, loss of duty time, hospitalization, and an increased risk of attrition [8–11]. Various types of preventive training programs have been developed to decrease the amount of LE injuries in the military [12–18]. An effective preventive training program type is postural stability training [14]. Preferably, these training programs are offered to servicemen who demonstrate insufficient postural stability, as they are at increased risk of developing LE (re)injuries [19, 20]. To identify servicemen suspected of having insufficient postural stability, an objective measurement of postural stability is needed. However, to the best of our knowledge, a standardized, reliable method for measuring postural stability in the military is lacking.

Several methods exist to quantify postural stability [21–23]. A commonly used method is the measurement of center of pressure (COP) excursions using a force plate [24–28]. One systematic review by Ruhe et al. [29] and a study by Doyle et al. [30] offered recommendations for maximizing the reliability of COP measurements. These studies were mostly conducted in healthy participants, but not in a military population. It is arguable whether the results of these studies can be generalized to servicemen [31]. Many military functions require above-average levels of physical strength and endurance. In contrast to civilian jobs or professional sports, the daily tasks of servicemen often consist of repetitive heavy lifting and carrying, marching and driving [32].

Several aspects of COP measurements, such as the measurement duration and number of repetitions, have been shown to influence the reliability [29, 33, 34]. Additionally, there are indications that visual feedback of the performance during a stability task leads to better postural stability in healthy adults and in patients with Parkinson’s disease, but the effects of visual feedback on postural stability and intrasession reliability in a military population is unknown. In addition to the effect of feedback on intrasession reliability, little is known about the effect of the foot position on this outcome. According to the systematic review by Ruhe et al. [29], only one study evaluated the effect of the foot position (small and normal stance) on the intrasession reliability of postural stability measurements [35].

In addition, it is unknown whether the results of a measurement in a given foot position can predict the results of a measurement in another foot position, which could considerably reduce the testing time.

Given the high burden of LE injuries in the military, as well as the need for an objective measurement of postural stability, we designed a study with the primary aim of examining the influence of the number of repetitions, foot position, and real-time visual feedback on postural stability and their effects on intrasession reliability [8–11]. The secondary objective of this study was to assess the concurrent validity of the measurements in different foot positions.

We hypothesized that postural stability, in terms of the COP velocity (COPv), will improve when real-time feedback on the COP velocity is provided, especially in the wide foot position, as the measurements are easier to perform, and participants might be less attentive in this position. We had no a priori hypotheses concerning the effect of the foot position or the use of real-time feedback on the reliability of COP velocity measurements.

Knowledge about the intrasession reliability and concurrent validity of COP measurements in different foot positions can contribute to the development of a reliable standardized protocol for the measurement of postural stability in servicemen.

## Methods

This quantitative, cross-sectional study was conducted according to the principles of the Declaration of Helsinki [36]. The protocol of this study was approved by the Medical Research Ethical Council (METC) Brabant (reference NW2018–22) and the DGO (reference DGO141117021).

### Participants

From December 2017 until February 2018, servicemen were recruited from different military bases in the Netherlands using posters and by word of mouth. Participants were included if they were between 20 and 50 years of age, healthy, in active duty and had sufficient language skills to understand the instructions. The exclusion criteria were recent LE injury (< 1 year) and any condition that might interfere with stability (such as current LE pain or neurological disorders). Participants were asked not to perform sports activities for 12 h prior to the measurements, since fatigue could influence the outcome measurements [37]. All participants signed an informed consent before the measurements started.

### Equipments

**Fig. 1:**
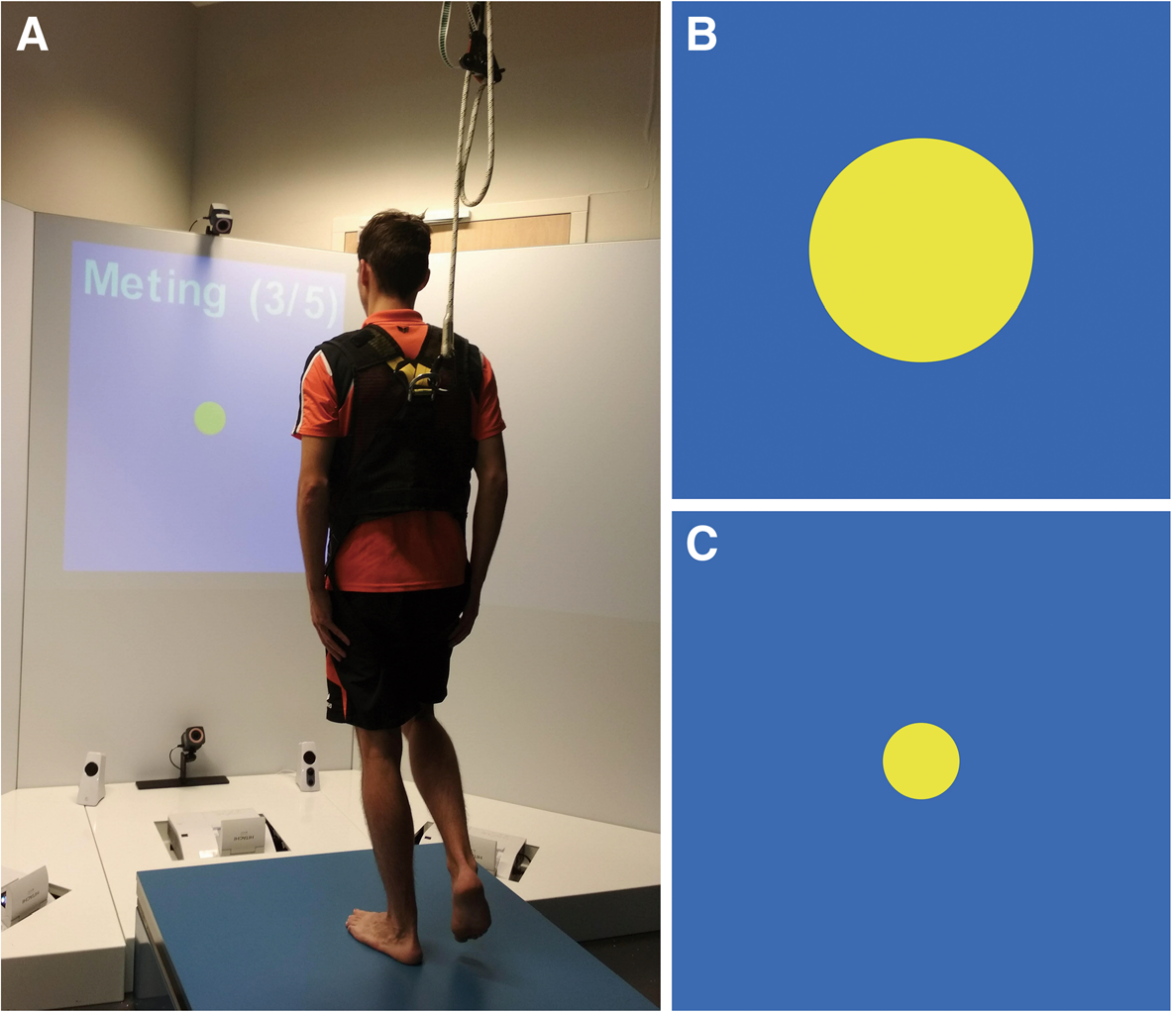
Test setup and visual feedback. **a**: Test setup. **b**: Visual feedback when the mCOPv was high (postural instability). **c**: Visual feedback when the mCOPv was low (postural stability). mCOPv: Mean velocity of the Center of pressure

Data were collected at the Military Rehabilitation Center (MRC) Aardenburg in Doorn, the Netherlands. All participants visited the MRC on one occasion for testing. Testing was performed on a 100 cm (cm) × 100 cm force plate, which is part of the Dynamic Stability and Balance Learning Environment (DynSTABLE, Motek Forcelink BV, The Netherlands). Participants stood 180 cm in front of a 120 cm × 120 cm screen that displayed the real-time visual feedback, which was represented by a yellow circle that became larger when postural stability decreased and smaller when postural stability increased (Fig. 1).

The diameter of the circle (in cm) was calculated as: *10 + 3* × *COPv.* COPv was expressed in (cm/s).

For example, the circle diameter was 13 cm if the actual COPv was 1 cm/s and 25 cm if the actual COPv was 5 cm/s.

During the measurements, participants wore a safety harness (Petzl® Newton Fast Jak, Crossel,France). The safety harness was suspended overhead to prevent the participants from falling, but no weight support was provided.

### Data collection

The demographic information collected included age, sex, height, weight, and body mass index. The position of the COP was filtered online with a unidirectional low-pass second-order Butterworth filter with a cut-off frequency of 1 Hz and sampled with 100 samples per second. This method has been shown to result in good intrasession reliability in bipedal COP measurements [29]. After each measurement, the mean velocity of the COP (mCOPv) was calculated and saved. According to a review by Ruhe et al. [29], COP summary measurements should be used to decrease the extreme effects of individual extreme readings. In addition, the mCOPv is one of the most commonly used COP parameters, and can be considered the most reliable traditional COP parameter for measuring postural stability [29].

### Procedures

The testing protocol was explained to participants, and they were allowed time to become familiar with the force plate. Thereafter, the participant completed the measurements.

All measurements were performed with bare feet. Participants were asked to stand as still as possible and to keep their arms against their body during the measurements [29]. The measurements included three different foot positions: wide stance (feet placed at shoulder width, lateral malleoli under the shoulders), small stance (medial malleoli against each other), and on one leg (the nondominant leg of each participant was measured. The dominant leg was defined as the leg with which the participant preferred to kick a football). An experienced physiotherapist instructed the participants and checked the position of the participants during each measurement. The stability of each foot position was evaluated with and without real-time visual feedback of the mCOPv, resulting in 6 trials.

During each trial, the mCOPv was measured five consecutive times, for 45 s each time, with a 15-s rest between measurements. A measurement duration of 45 s has been shown to result in good intrasession reliability (*r* = 0.84) in bipedal COP measurements [34]. Longer trial durations could negatively influence the reliability of measurements on one leg as a result of fatigue [38, 39]. Averaging multiple COP measurements in one testing session leads to more reliable data compared to conducting one measurement [28, 29, 40]. Averaging three to five measurement repetitions should be appropriate to gain good intrasession reliability (*r* > 0.75) [29]. The order of the foot positions and whether or not visual feedback was displayed was randomized. Between the different foot positions, a rest period of 1 min was provided.

### Statistical analysis

The data analysis was performed using SPSS Version 24 (SPSS Inc., Chicago IL, USA). The alpha level was set at 0.05 for all statistical analyses.

To determine the influence of the number of repetitions, foot position and visual feedback on the mCOPv, as well as their interactions, a generalized estimating equation (GEE) was used. No significant interactions were removed from the model.

To assess the number of measurements needed to obtain good relative reliability, intraclass correlation coefficient (ICC) estimates were calculated using a two-way mixed-effects model with absolute agreement (as described by McGraw and Wong) [41] based on a mean rating of the first two, the first three, the first four, and all five repetitions of the measurements. For the calculation of the ICC of a single trial, we used a two-way mixed-effects model with absolute agreement based on single measurements, using the data from the first two trials of each participant. If the GEE showed a significant effect of foot position and/or feedback on the mCOPv, ICCs were calculated for these conditions separately.

As recommended by Koo and Li, the lower bounds of the 95% confidence interval (CI) of the ICC estimates were used as the basis to evaluate the level of reliability [42]. Lower bounds between 0.00 and 0.50 were defined as having poor reliability, between 0.50 and 0.75 indicated moderate reliability, 0.75 to 0.90 indicated good reliability, and lower bounds > 0.90 were defined as having excellent reliability [42]. We considered a lower bound of the 95% CI of the ICC of ≥0.75 as sufficient for the recommendation of the number of repetitions.

The standard error of measurement (SEM) was calculated to obtain absolute reliability. The SEM quantifies the reliability of scores within individual participants on different occasions [43]. The SEM was calculated for each possible combination of foot position and number of repetitions using the formula: $SEM= SD\sqrt{1- ICC}$[44]*,* in which SD was the standard deviation between subjects (each subject value was calculated by averaging the mean center of pressure velocity scores over the specified number of repetitions of that foot-position).

Since there is no definition of “adequate” absolute reliability, we refrained from labeling these values as poor, moderate, good, or excellent [45]. With the SEM, it is possible to calculate the 95% CI for an individual on a second test occasion (assuming that stability has not changed between tests) as follows: *Lower limit = mCOPv* − *(1.96* × *SEM), and upper limit = mCOPv* + *(1.96* × *SEM).* If the participant’s score is below this lower limit on a retest, it is likely that the stability of the individual has improved.

To assess concurrent validity between the different foot positions, Pearson correlation coefficients were calculated for each combination of the three foot positions. Pearson correlations were interpreted as follows: 0.00 to 0.30 negligible correlation, 0.30 to 0.50 low correlation, 0.50 to 0.70 moderate correlation, 0.70 to 0.90 high correlation, and 0.90 to 1.00 very high correlation [46]. The coefficient of determination (i.e., *R*^*2*^) was presented to show the proportion of the variance of the mCOPv value that was predictable by the foot position. *R*^*2*^ values of ≥0.75, indicating a Pearson correlation coefficient of ≥0.866, were considered to be substantial [47]. That is, if *R*^*2*^ was ≥0.75 between two measurements in different foot positions, the result of a measurement in one-foot position was considered to be able to predict the result of the measurement in the other foot position.

## Results

Twenty servicemen participated in the study. Demographic information of the participants can be found in Table 1. All participants completed the measurements.

**Table 1:** Descriptive characteristics of the studied population (*N =* 20)

Variable	Mean(SD)
Age (year)	28.1(6.9)
Height (cm)	178.5(8.8)
Weight (kg)	77.6(12.1)
Body mass index (kg/m^2^)	24.3(2.8)

*SD* Standard deviation

### Effect of repetition number, foot position and visual feedback on mCOPv measurements

The mean mCOPv scores and their standard deviations are displayed in Table 2, and the results of the GEE are shown in Table 3. The lowest mCOPv values were found in the wide-stance measurements, followed by the small-stance measurements and measurements on one leg. There was a significant main effect of foot position (*p* < 0.001), but not of visual feedback (*p* = 0.119) or repetition number (*p* = 0.915). None of the interaction terms were significant, and they were therefore removed from the model.

**Table 2:** mCOPv measurements for all foot positions, with and without visual feedback [*N* = 20, Mean(SD)]

Variable	Measurement 1	Measurement 2	Measurement 3	Measurement 4	Measurement 5
Wide stance, with feedback	2.30 (0.66)	2.50 (0.83)	2.54 (0.77)	2.71 (0.99)	2.50 (0.95)
Small stance, with feedback	4.85 (1.23)	5.30 (2.85)	4.97 (1.48)	4.79 (1.40)	4.68 (1.19)
One leg, with feedback	10.83 (4.76)	10.37 (2.97)	11.32 (7.30)	10.76 (2.76)	9.94 (1.93)
Wide stance, without feedback	2.67 (0.80)	2.80 (0.93)	2.83 (1.03)	2.73 (0.87)	2.64 (0.87)
Small stance, without feedback	4.83 (1.21)	4.92 (1.23)	4.87 (1.64)	5.11 (1.41)	5.09 (1.42)
One leg, without feedback	11.27 (4.65)	10.66 (4.65)	11.20 (5.92)	10.48 (3.92)	12.06 (7.07)

*SD* Standard deviation, *mCOPv* Mean velocity of the center of pressure

**Table 3:** Effects of repetitions, foot position, and feedback on mCOPv

Item	*β* (95% CI)	*P*value
Repetitions	0.005 (−0.085, 0.095)	0.915
Foot position		
Wide	2.734 (2.211, 3.256)	< 0.001*
Small	5.053 (4.613, 5.493)	< 0.001*
One leg	11.003 (9.013, 12.993)	< 0.001*
Feedback	−0.254 (−0.573, 0.065)	0.119

*Significant difference (*P* < 0.05); *β*. beta coefficient; mCOPv. Mean velocity of the center of pressure

### Intrasession reliability for each foot position

Since the foot position significantly affects the mCOPv score, ICCs were calculated separately for each foot position.

**Fig. 2:**
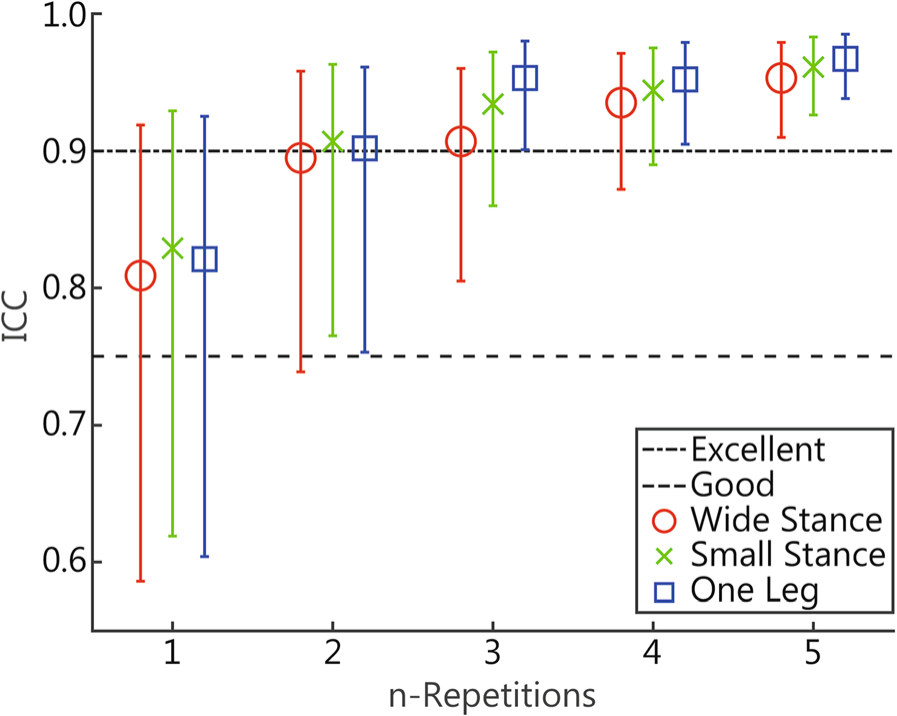
ICC estimates and 95% CIs if measured one, two, three, four, and five times. The lowest dotted line represents the minimum desired ICC

The ICC estimates, their 95% CIs and the SEM of the ICC of each foot position can be found in Table 4. The ICC estimates and their 95% CIs are represented graphically in Fig. 2.

**Table 4:** ICC estimates and the SEM for mCOPv measurements in all foot positions

	1 repetition^a^			2 repetitions^b^			3 repetitions^b^			4 repetitions^b^			5 repetitions^b^		
	ICC	95% CI	SEM	ICC	95% CI	SEM	ICC	95% CI	SEM	ICC	95% CI	SEM	ICC	95% CI	SEM
Wide stance	0.809	0.586, 0.919	0.378	0.895	0.739, 0.958	0.281	0.907	0.805, 0.960	0.280	0.935	0.872, 0.971	0.231	0.953	0.910, 0.979	0.195
Small stance	0.829	0.619, 0.929	0.505	0.907	0.765, 0.963	0.372	0.934	0.860, 0.972	0.349	0.944	0.890, 0.975	0.325	0.961	0.926, 0.983	0.273
One leg stance	0.821	0.604, 0.925	2.760	0.902	0.753, 0.961	2.042	0.953	0.901, 0.980	1.370	0.952	0.905, 0.979	1.253	0.967	0.938, 0.985	1.088

*ICC* Intraclass correlation coefficient, *CI* Confidence interval, *SEM* Standard error of measurement; ^a^Single measurement; ^b^Average measurements; mCOPv: Mean velocity of the center of pressure

The ICC estimates of the wide-stance measurements varied from 0.895 (the first two repetitions) to 0.953 (five repetitions). Based on the lower bounds of the 95% CIs, the intrasession reliability of the wide-stance measurements was moderate if the measurements were conducted twice. If the measurements were conducted three or four times, the intrasession reliability was good. If the measurements were performed five times, the intrasession reliability was excellent.

For the small-stance measurements, the ICC estimates varied from 0.907 (the first two repetitions) to 0.961 (five repetitions). Based on the lower bounds of the 95% CIs, the intrasession reliability of the measurements was good if the measurements were conducted two, three, or four times. If the measurements were conducted five times, the intrasession reliability was excellent.

The ICC estimates of the one-leg stance measurements varied from 0.902 (the first two repetitions) to 0.938 (five repetitions). Based on the lower bounds of the 95% CIs, the intrasession reliability of the measurements was good if the measurements were performed two times and excellent if the measurements were conducted three, four, or five times.

The SEM values for measurements without visual feedback were 0.280 (wide-stance measurements if conducted three times), 0.372 (small-stance measurements if conducted two times), and 2.042 (measurements on one leg if conducted two times). This means that if an individual scored 5.0 cm/s in the wide foot position (3 × 45 seconds) on one test occasion, his or her score would significantly change if below 4.451 (5–0.280 * 1.96) or above 5.549 (5–0.280 * 1.96) on the next test occasion.

### Concurrent validity

A significant moderate correlation (*r* = 0.642) was found between the wide-stance and small-stance measurements. Between the small-stance and one-leg measurements, a significant low correlation (*r* = 0.457) was found. The correlation between the wide-stance and one-leg measurements was found to be negligible (*r* = 0.090). *R*^2^ values were all below 0.75, which means they were considered to be not substantial (Table 5).

**Table 5:** Pearson correlations among the three foot positions

Position	r	R^2^	*p*
Wide stance and small stance			
Without feedback	0.642	0.412	0.002*
Wide stance and one leg stance			
Without feedback	0.090	0.008	0.705
Small stance and one leg stance			
Without feedback	0.457	0.209	0.043*

*Significant difference (*P* < 0.05); *β*: beta coefficient; *r*: Pearson product-moment correlation coefficient; *R*^2^: coefficient of determination

## Discussion

To the best of our knowledge, this is the first study to examine the influence of foot position, visual feedback, and the number of repetitions on the mCOPv, as well as their effects on intrasession reliability.

### Impact of foot position, number of measurements, and visual feedback on mCOPv

#### Foot position

Our data show that the mCOPv increased in the small-stance and one-leg measurements compared with the measurements in a wide foot position. This finding is in agreement with previous studies, since it is widely assumed that postural stability decreases in more challenging foot positions [48–51].

#### Number of measurements

Our results are also in accordance with those reported in studies by Doyle et al. [30]. and Golriz et al. [52], who both found that mCOPv values were not considerably affected by the number of measurements.

#### Visual feedback

Regarding the visual feedback, our results differ from those reported in a study by Boudrahem et al. [53], who found a significant effect of visual feedback. In their study, healthy adults performed better with feedback than without feedback. Rougier et al. also found that providing feedback led to better postural control among healthy adults, whereas we did not observe an effect of visual feedback [54]. This difference could be due to the different populations in which the measurements were conducted; healthy participants were tested in the studies by Boudrahem et al. [53] and Rougier et al. [54], whereas we measured servicemen.

### Impact of number of measurements and foot position on reliability

#### Number of measurements

In regard to the relative intrasession reliability, the lower bounds of the 95% CIs of the wide-stance measurements were considered to have good reliability if the measurements were conducted three times or more. For the small-stance measurements and measurements on one leg, intrasession reliability was considered to be good if the measurements were conducted two times or more.

The results of a study by Lafond et al. [28], who examined the number of repetitions and time frame required to yield good intrasession reliability of wide-stance COP measurements, are largely in accordance with our results. Lafond et al. examined the intrasession reliability of mCOPv measurements in healthy, elderly individuals and found that three wide-stance measurements of 30 s (a total of 90 s) leads to an ICC > 0.90, whereas we found an ICC of 0.895 when measurements were performed two times for 45 s (also a total of 90 s). In addition, they found that two wide-stance measurements of 120 s (a total of 240 s) were required to reach an ICC of > 0.90, whereas we found excellent ICC estimates if wide-stance measurements were performed three times for 45 s (a total of 135 s). These small differences could have been due to the studied population (elderly versus servicemen) or the sampling duration (120 s versus 45 s in our study). Furthermore, our results are in accordance with the recommendations from a systematic review by Ruhe et al. [29], who concluded that averaging three to five trials should be appropriate to yield good reliability. However, for the small-stance measurements and measurements on one leg, we concluded that two measurements should be appropriate to yield good reliability.

#### Foot position

A study by Hill et al. [35], who compared the effects of different foot positions in COP measurements on intrasession reliability, found that small-stance measurements led to lower intrasession reliability compared to wide-stance measurements, whereas we found higher ICC values for small-stance measurements compared with wide-stance measurements (for all numbers of repetitions). In addition, Hill et al. [35] found poor intrasession reliability for small-stance measurements and moderate intrasession reliability for wide-stance measurements. This is in contrast to our findings, since we found good intrasession reliability when the measurements were conducted three times for wide-stance measurements and two times for small-stance measurements. Again, these differences could have been due to the sampling duration in their study (25 s versus 45 s in our study) and differences in the studied population (elderly versus servicemen). In addition, Hill et al. reported the dispersion index as a measure of postural stability, whereas we measured the mCOPv, which is a more reliable parameter for measuring postural stability [29].

### Concurrent validity

Regarding concurrent validity, the present study showed a moderate correlation between wide- and small-stance measurements. Between small-stance and one-leg measurements, significant low correlations were found. Correlations between wide-stance and one-leg measurements were found to be negligible. *R*^2^ values were all less than 0.75, which means they were considered to be not substantial.

These results indicate that the mCOPv value of a measurement in a particular foot position cannot predict the mCOPv value of a measurement in another foot position. To our knowledge, this study is the first to focus on concurrent validity in different foot positions. Therefore, a comparison with the literature appeared to be impossible.

### Limitations

This study has several limitations that need to be addressed. First, we could not include a flowchart of the sampling process, since we sampled participants using posters that were spread across different military bases and by word of mouth. Consequently, this could have led to selection bias, as more fit or motivated servicemen might have signed up for the study. This could have also led to lower mCOPv values (i.e., better postural stability) and a smaller between-subject variance compared with the actual population, possibly resulting in an overestimation of the mCOPv scores and an underestimation of the ICC values. Furthermore, our study included 16 men and 4 women, which could have led to an underestimation of the mCOPv values, since it is known that female servicemen outperform male servicemen in postural stability [55]. However, the percentage of women in our sample contained approximately as many women as the percentage of women working in the Dutch military. Therefore, we consider this sample to be representative in terms of the number of males and females. In line with this limitation, we did not present the results by age group. Most of the participants (17) were 22 to 35 years of age, and three of them were 40 to 45 years of age. It is likely that inclusion of the elderly participants might have caused lower mCOPv values, since it has been demonstrated that postural stability declines with age [56].

Second, the type of feedback (real-time visual feedback represented on a screen as a yellow circle that becomes larger when postural stability decreases) we used could have led to the fact that we did not find an effect of feedback on postural stability, since it is known that the type of visual feedback (e.g., internal/external feedback, with or without delay) influences postural stability.

Another potential limitation was the variation in foot positions when the participants stepped off and back onto the force platform during the rest periods. Although an experienced physiotherapist checked the position of the feet in every measurement, the exact foot position might not have been the same in all measurements, which could therefore have influenced the results. However, this method is frequently used in daily practice.

Finally, our study focused only on postural stability in different foot positions (static stability). Dynamic measurements of postural stability might be more relevant for servicemen, since they are more challenging and may better differentiate between risk factors [57]. Moreover, the work content of servicemen requires great dynamic stability [58].

### Recommendations

To yield good intrasession reliability, we recommend performing measurements in a wide foot position three or more times for 45 s, and in a small foot position and on one leg two or more times for 45 s. Since we found the mCOPv values to be roughly the same over the sessions, we do not recommend the use of a familiarization session, as this would probably not influence the mCOPv.

Depending on the foot position of interest, measurements should be conducted in that particular foot position. For example, if a clinician is interested in stability on one leg (e.g., in athletes), measurements should be performed with participants in a one-leg stance. If stability in a wide stance is more interesting (e.g., shortly after a stroke), the measurements should take place in a wide foot position.

This study is a first step in developing a reliable and valid protocol for the measurement of postural stability in servicemen. In future research, a next step could be to test this protocol in a cohort of servicemen who are more likely to have insufficient postural stability, for example, servicemen with LE injuries, to determine normal values and cut-off scores for mCOPv data. These cut-off scores could aid in the identification of other servicemen with insufficient postural stability who might benefit from preventive training programs. Furthermore, to identify differences in postural stability over time, for example, before and after a training program or injury, intersession reliability should be examined in further research. Given the relevance for dynamic stability in servicemen, we recommend that further research should also focus on dynamic measurements. It is possible that a shorter time series (e.g., 30 s) would also yield good intrasession reliability [59], which could be examined in further research as well.

## Conclusions

Postural stability decreases in more challenging foot positions, but no effects of visual feedback or the number of repetitions on the mCOPv were found. To yield good intrasession reliability, measurements in a wide foot position should be conducted three or more times for 45 s, and in a small foot position or on one leg, the measurements should be conducted two or more times for 45 s. Since concurrent validity between the different foot positions was found to be moderate or worse, we recommend performing measurements in the foot position that is the most relevant for the servicemen of interest.

This reliable measurement protocol can be used to identify differences in postural stability between both legs and to obtain an overall indication of postural stability in servicemen. Caution is needed when generalizing our findings to other populations.

## Data Availability

The datasets used and/or analyzed during the current study are available from the corresponding author upon reasonable request.

## Abbreviations

COP: Center of pressure
COPv: COP velocity
DGO: Defense Health Organization
DynSTABLE: Dynamic Stability and Balance Learning Environment
GEE: Generalized estimating eq.
ICC: Intraclass correlation coefficient
kg: kilograms
LE: Lower extremity
m: meters
mCOPv: mean velocity of the COP
METC: Medical Research Ethical Council
MRC: Military Rehabilitation Center
